# Probing Rate-Dependent Liquid Shear Viscosity Using Combined Machine Learning and Nonequilibrium Molecular Dynamics

**DOI:** 10.1021/acs.jctc.5c00293

**Published:** 2025-06-03

**Authors:** Hongyu Gao, Minghe Zhu, Jia Ma, Marc Honecker, Kexian Li

**Affiliations:** † Department of Materials Science & Engineering, 9379 Saarland University, Campus C6.3, 66123 Saarbrücken, Germany; ‡ School of Civil and Environmental Engineering, 12418 Changsha University of Science and Technology, Changsha 410114, PR China

## Abstract

Accurately measuring liquid dynamic viscosity across a wide range of shear rates, from the linear-response to shear-thinning regimes, presents significant experimental challenges due to limitations in resolving high shear rates and controlling thermal effects. In this study, we integrated machine learning (ML) with nonequilibrium molecular dynamics (NEMD) simulations to address these challenges. A supervised artificial neural network (ANN) model was developed to predict viscosity as a function of shear rate, normal pressure, and temperature, effectively capturing the complex interplay among these variables. The model reveals distinct trends in shear viscosity, characterized by the shear-thinning exponent, and highlights nonmonotonic behavior in the radius of gyration components, reflecting molecular morphological changes driven by rate-dependent volume expansion. Notably, temperature effects diminish at higher shear rates, where molecular alignment and spacing dominate the response to shear. By implementing the ‘fix npt/sllod’ command in LAMMPS, we achieve precise constant-pressure control in NEMD simulations, ensuring accurate representation of system dynamics. This study demonstrates the potential of ML-enhanced NEMD for efficient and accurate viscosity prediction, providing a robust framework for future research in complex fluid dynamics and material design.

## Graphical abstract

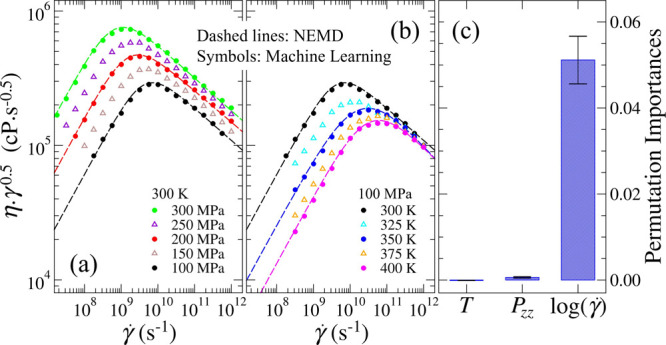

## Introduction

Dynamic viscosity (η), defined as the ratio of shear stress (τ) to shear rate (γ̇): η = τ/γ̇, quantifies a fluid’s resistance to deformation under shear. In the linear-response regime at low γ̇, shear stress is proportional to the shear rate (τ ∝γ̇), resulting in constant viscosity known as Newtonian viscosity (η_0_). Beyond a critical shear rate (γ̇_0_), many liquids, including colloidal suspensions and polymer melts, exhibit shear-thinning behavior,^1^ where viscosity decreases with increasing γ̇. The functional form of this transition—whether logarithmic (as described by the Eyring theory^2^) or following a power-law (as in the Carreau model^3^)—and its sensitivity to measurement protocols have been long debated.^4–7^ These models offer valuable frameworks but also underscore the importance of the experimental context when interpreting flow behavior. Shear-thinning occurs when the deformation time scale (1/γ̇) approaches or surpasses the fluid’s structural relaxation times. In polymers, this often reflects chain alignment and disentanglement, while in colloidal systems it arises from flow-induced microstructure changes.^8–10^ This leads to a reduced resistance to flow and a corresponding drop in viscosity.

Probing the viscosity across a wide range of γ̇ presents significant challenges in both experiments and simulations. Experimental techniques, such as tribometry and viscometry,^4^ struggle at high γ̇ due to thermal heating effects,^11^ which cause volume expansion^12^ and potential underestimation of η. Nonequilibrium molecular dynamics (NEMD) simulations^10^ offer precise control over parameters like temperature (*T*), normal pressure (*P*_*zz*_), and shear rate, providing an alternative for studying viscosity. However, NEMD simulations face limitations, including long runtimes required to achieve sufficient signal-to-noise ratios at low γ̇, thereby making it difficult to accurately predict Newtonian viscosity for highly viscous systems. Additionally, the accuracy of NEMD predictions depends on factors such as the choice of molecular model (explicit or coarse-grained), force field reliability,^13^ and thermostatting methods.^14^ Reconciling experimental and simulation results across overlapping γ̇ regimes remains challenging, particularly for liquids with complex molecular structures or under extreme conditions.

Data-driven machine learning (ML) approaches have emerged as powerful tools for predicting material properties and accelerating material design.^15–17^ For instance, physics-informed quantitative structure–property relationship (QSPR) models,^18,19^ utilizing descriptor-based or graph-based neural networks, have successfully predicted kinematic viscosity, reducing experimental costs. When integrated with molecular dynamics (MD) simulations,^20,21^ these models achieved enhanced predictive accuracy. However, ML models for dynamic viscosity remain scarce^20,22,23^ due to limited data sets encompassing diverse liquid types and conditions, as well as challenges in extrapolating beyond training data. Experimental acquisition of domain-specific knowledge is often resource-intensive, highlighting the importance of computational simulations with physical interpretability. Dynamic viscosity, η­(γ̇), under varying *T* and *P*_*zz*_ conditions often follows time–temperature–pressure superposition (TTPS),^24,25^ which enables data normalization onto a master curve.^1,5^ However, implementing TTPS requires prior knowledge of η_0_ and γ̇_0_, which are themselves often targets of prediction, adding complexity to the process.

In this work, we developed an ML model to predict the rate-dependent dynamic viscosity of *n*-hexadecane, a representative liquid, by integrating all-atom NEMD simulations. Our model, based on an artificial neural network (ANN), is trained on NEMD simulation data and validated for bulk-phase liquids, distinguishing it from studies of confined fluids in nanoscale slits,^26,27^ where oscillatory density profiles typically arise. By emphasizing the importance of constant-pressure control, we systematically explored the dependence of η on *T* and *P*_*zz*_ in the shear-thinning regime, capturing the interplay between molecular dynamics and macroscopic flow behavior. Despite challenges posed by limited training data, our approach demonstrates robust predictive accuracy and computational efficiency, providing a scalable framework for dynamic viscosity prediction. This work is expected to advance the understanding of fluids’ shear-thinning behavior and to establish a foundation for extending ML-driven approaches to more intricate systems and extreme conditions.

## Methodology

### Molecular Dynamics

Both nonequilibrium molecular dynamics (NEMD) and equilibrium molecular dynamics (EMD) simulations were conducted using the open-source code LAMMPS,^28^ with *n*-hexadecane selected as the model system due to prior experience^10,29^ and computational efficiency. The simulation cell contained 100 *n*-hexadecane molecules, with dimensions (∼4 nm minimum length) validated in advance to exceed 30× the persistence length (∼0.13 nm) and 10× the maximum radius of gyration (*R*_g,max_ ≈ 0.37 nm). Finite-size effects were confirmed to be negligible by comparing with simulations using 200 molecules, which showed <2% variation in both η and *R*_g_. An all-atom representation was employed to ensure accurate shear stress calculations, as preliminary tests using the united-atom TraPPE force field^30^ revealed a ∼20% underestimation. Interatomic interactions were described using the L-OPLS^31,32^ force field, which is optimized for long-chain alkanes, incorporating bonded (bond stretching, angle bending, and torsion) and nonbonded (van der Waals, Coulombic) terms. EMD simulations primarily validated Newtonian viscosity via the Green–Kubo method^33,34^ (see the Supporting Information for details), while references to “simulation results” default to NEMD unless otherwise specified.

Shear viscosity was calculated as the ratio of shear stress (τ) to shear rate (γ̇) under linear planar (Couette) flow conditions, where the velocity gradient perpendicular to the shear plane remains constant. This approach avoids the molecular layering effects associated with solid wall-induced boundary-driven shear,^26^ thereby providing a true representation of bulk liquid viscosity. Lees–Edwards^35^ equivalent periodic boundary conditions (PBC) were applied to remap atom positions and velocities crossing the simulation boundaries, while the standard SLLOD algorithm^36^ was employed to model both conservative and dissipative forces. Simulations were performed under the NPT ensemble, regulated by the Nosé–Hoover^37,38^ thermostat and barostat to maintain system temperature and pressure. The choice of NPT/SLLOD over NVT/SLLOD is justified in the Results section. Shear rate, temperature, and normal pressure (*P*_*zz*_) were varied across ranges of 10^8^–10^12^ 1/s, 300–400 K, and 100–300 MPa, respectively, with a simulation time step of 1 fs. Error bars were calculated from uncorrelated data achieved, with the dump frequency optimized via the time-autocorrelation function (*t*-ACF) analysis.

### Machine Learning

A machine learning (ML) model was developed using an artificial neural network (ANN) regressor trained on data derived from nonequilibrium molecular dynamics (NEMD) simulations. The input features included applied conditions: shear rate (γ̇), temperature (*T*), and normal pressure (*P*_*zz*_), with the target output being liquid shear viscosity (η). The ANN was selected as the final regression algorithm due to its superior performance compared to other tested models, including linear regression, random forest, extra trees, gradient boosting, support vector machines, and *k*-nearest neighbors. Performance metrics for various regression algorithms are provided in the SI (Table S1), demonstrating that the ANN achieves the best overall predictive accuracy. Ensemble methods such as voting and stacking regressors were also evaluated but did not outperform the ANN. To capture rate-dependent structural and thermodynamic factors, additional variables such as density (ρ) and radius of gyration components (*R*_g_^*x*^, *R*_g_^*y*^, and *R*_g_^*z*^) were incorporated into the input features. However, their contributions to prediction accuracy were found to be marginal, suggesting that the primary input features (γ̇, *T*, and *P*_*zz*_) were sufficient for accurate viscosity prediction.

The data set was randomly split into 80% for training and 20% for validation to ensure unbiased evaluation of model performance. To address limited data dimensionality, five replicate results from parallel simulation runs were included for each unique set of conditions (γ̇, *T*, *P*_*zz*_), enhancing the model’s ability to capture variability and improve generalization. In total, 50 distinct combinations of conditions were sampled, resulting in 250 sets of training inputs. Logarithmic transformations were applied to γ̇ and η to account for their broad value ranges and inherent nonlinear relationships. The models were implemented by using the scikit-learn and Keras libraries. The ANN architecture consisted of a feed-forward network with two hidden layers (64 and 32 neurons, respectively), utilizing the ReLU activation function and the Adam optimizer for efficient training convergence. Hyperparameter optimization was conducted via randomized search combined with 5-fold cross-validation to ensure robust parameter selection. The validity of the ML model was benchmarked against the NEMD results under identical conditions. Model performance was comprehensively evaluated using metrics such as mean squared error (MSE), mean absolute error (MAE), *R*-squared (*R* ^2^), and root mean squared error (RMSE), proving a thorough evaluation across the varying scales of η. Further validation, including fitting to the Carreau–Yasuda model, is detailed in the Results section.

## Results and Discussion

### Running NEMD under an NPT Ensemble

In the shear-thinning regime, molecules moving rapidly along the streaming direction lack sufficient time to relax and dissipate energy, arising from intense atomic collisions and internal friction. Although excess heat can be removed via velocity rescaling, molecular morphological changes under constant normal pressure (*P*_*zz*_) result in system volume expansion and a corresponding drop in density (ρ), as illustrated in the inset of Figure 1b. This phenomenon leads to an overestimation of τ at high γ̇ under constant density (const.-ρ) control due to elevated hydrostatic pressure. To address this, we modified the LAMMPS source code to implement a new command, ’fix npt/sllod‘, which enables barostat control exclusively in the normal direction while incorporating the SLLOD algorithm to handle in-plane cell deformation. By regulating only *P*_*zz*_, this approach avoids conflicts with imposed in-plane domain deformation, where atom positions and velocities are remapped based on the Lees-Edwards periodic boundary conditions (PBC).^39^ This modification enhances physical fidelity, maintains system stability during nonequilibrium dynamics, and significantly improves modeling efficiency compared to pressure interpolation methods (see SI for details). The modified C++ source code is provided in the Supporting Information.

**Figure 1:**
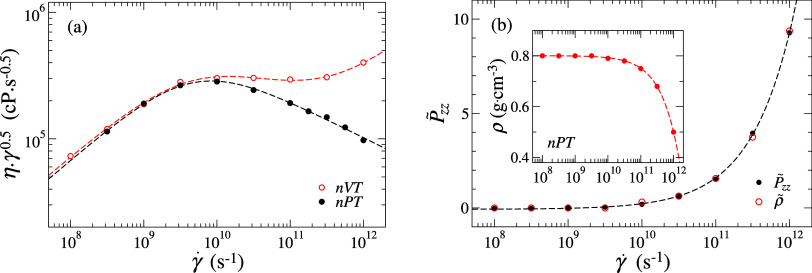
(a) Intermediate scaling plot of shear viscosity (η) as a function of shear rate (γ̇) from constant-density (ρ) and constant-normal-pressure (*P*_*zz*_ = 100 MPa) simulations at temperature *T* = 300 K. (b) Collapse of the normalized density (ρ̃) onto the normalized *P̃*_*zz*_ curve as a function of γ̇ with a proportionality constant α = 2.5. The inset shows the variation of ρ with γ̇ under const.-*P*_*zz*_ control.

Predictions from const.-ρ (NVT control) and const.-*P*_*zz*_ (NPT control) are compared through intermediate scaling plots,^10^ where data are fitted using the Carreau–Yasuda (CY) model:^3,40^

$$\eta \left(\mathrm{\gamma ̇}\right)=\eta_{\infty }+\left(\eta_{0}-\eta_{\infty }\right){\left[1+{\left(\frac{\mathrm{\gamma ̇}}{{\mathrm{\gamma ̇}}_{0}}\right)}^{a}\right]}^{n-1/a}$$ 1

where η_*∞*_ represents the second Newtonian viscosity, and *a* and *n* are fitting parameters defining the crossover curvature and shear-thinning exponent, respectively. As shown in Figure 1a, predictions in the linear response regime are comparable; however, differences grow beyond the crossover and become more pronounced at higher γ̇. Consistent with the findings of Daivis and Evans,^12^ η_*∞*_ is only necessary for const.-ρ predictions, serving as an artificial adjustment. The CY model demonstrates superior performance over the Eyring theory^2^ (as detailed in ref 10.), achieving a lower normalized logarithmic relative standard deviation, even when accounting for the parameter count (*N*_CY_ = 4 versus *N*_Eyring_ = 2).

In the Newtonian regime, the diagonal pressure tensor components (*P*_*ii*_, *i* = *x*, *y*, *z*) remain comparable to the isotropic hydrostatic pressure observed in bulk equilibrium in the absence of shear. However, as the strain rate increases and the system enters the shear-thinning regime, these pressures diverge, with *P*_*zz*_ exhibiting an exponential rise under const.-ρ conditions. Consequently, the relationship between pressure and density cannot be adequately described by traditional equations of state (EOS), such as the Tait^41^ or Murnaghan^42^ equations, due to shear-induced anisotropy.^10^ As shown in Figure 1b, the variation in normal pressure (*P̃*_*zz*_) under const.-ρ is proportional to the variation in density (ρ̃) under const.-*P*_*zz*_:

$${\mathrm{P̃}}_{zz}=\alpha \mathrm{\rho ̃}$$ 2

where *P̃*_*zz*_ = *P*_*zz*_^NVT^/*P*_*zz*_^NPT^ – 1, ρ̃ = 1 – ρ^NPT^/ρ^NVT^, and α is a proportionality constant. Nevertheless, the crossover observed in the γ̇ – ρ (or γ̇ – *P*_*zz*_) curves does not align with those in the γ̇ – η plots, indicating that changes in density (or normal pressure) do not fully explain the shear-thinning behavior. This highlights the need for further investigation into molecular morphology and microstructural changes under shear flow to better understand variations in shear stress.

### ML-Predicted Shear Viscosity

The NEMD data set, though limited in size, provides high-quality data derived from simulations averaged over sufficiently long times with a high signal-to-noise ratio. Using three input features, i.e., γ̇, *T*, and *P*_*zz*_, the ANN model demonstrates exceptional performance in reproducing the NEMD-predicted η across a wide range of γ̇ values and moderate variations in *T* and *P*_*zz*_. The {*T*, *P*_*zz*_, and γ̇} conditions used for training correspond directly to the solid symbols shown in Figure 2b. The model achieved an impressive MSE of 4.8 × 10^–4^, MAE of 1.6 × 10^–2^, and an *R* ^2^ score of 0.9991 on the training set, using a fixed random seed of 42. Training employed the Adam optimizer with a learning rate of 0.001, a batch size of 16, and 3000 epochs (these batch size and epoch values were selected as the best-performing configuration from a series of tests via hyperparameter tuning using a pipeline). Validation loss closely tracked the training loss, with both decaying exponentially with epochs, indicating effective and consistent learning. More details about the model performance are included in the SI. The small batch size may have introduced noise into the gradient updates, potentially helping the model explore the loss landscape more effectively and improving generalization performance. Alternative optimization algorithms, such as root-mean-square propagation (RMSProp) and Levenberg–Marquardt^43^ (LM), produced comparable results when tuned to their respective optimal hyperparameters.

**Figure 2:**
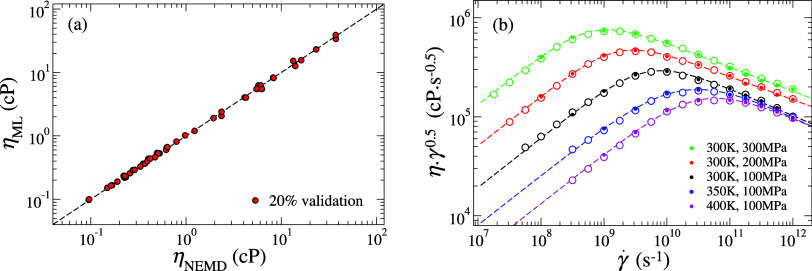
Comparison of shear viscosity (η) predicted by NEMD modeling and machine learning (ML) for (a) 20% validation data set and (b) additional untrained data at temperatures *T* ∈ {300, 350, 400 K} and normal pressures *P*_*zz*_ ∈ {100, 200, 300 MPa}. In (b), solid symbols denote NEMD predictions, hollow symbols represent ML predictions, and dashed lines indicate Carreau–Yasuda (CY) fits to the NEMD data. ML predictions are made with 95% confidence intervals.

The ML model demonstrates excellent predictive capability with validation results showing close agreement between predicted and NEMD-calculated viscosities (Figure 2a), confirming its reliability across the parameter space. Interpretability analysis confirms that the model captures physically meaningful trends. Permutation importance identified the logarithm of shear rate as the dominant predictor (importance score: 0.047 ± 0.003), consistent with its critical role in controlling non-Newtonian viscosity behavior. Normal pressure (0.012 ± 0.001) and temperature (0.008 ± 0.001) also contributed significantly, in line with known rheological dependencies. SHAP analysis reinforced these findings: shear rate displaced strong nonlinear influence reflecting shear-thinning, while temperature exhibited a negative correlation characteristic of Arrhenius-type behavior (SHAP: −2.97 ± 0.42). Normal pressure had a relatively modest impact (SHAP: −0.44 ± 0.12), consistent with its limited variation across the studied regime. Further analysis in the SI explores how feature importance shifts across shear- and thermally dominated regimes, and evaluates interaction effects between input variables.

To assess model confidence, we incorporated uncertainty quantification using Monte Carlo dropout with conservatively chosen rates (5–10%). This approach preserved high predictive accuracy (*R* ^2^ = 0.996) and provided physically meaningful uncertainty estimates, with a mean 95% confidence interval (CI) width of 0.19 log­(η) units. As detailed in the SI, uncertainty was elevated in regions of greater rheological complexity–namely, the shear-thinning crossover, low temperatures, and high normal pressures– demonstrating the model’s ability to recognize its own predictive limitations.

To further evaluate the model, predictions were performed under (*T*, *P*_*zz*_) conditions consistent with NEMD simulations, enabling direct comparisons through CY fitting. As shown in Figure 2b, the ANN model predictions (hollow symbols) not only reproduced the NEMD results (solid symbols) but also extended seamlessly along the CY fitting curves (dashed lines), achieving *an R* ^2^ of 0.99. Notably, the NEMD data in Figure 2b was not part of the training set, highlighting the model’s ability to generalize beyond the training data. We emphasize that the predictive accuracy of the model is largely influenced by the quality of the input data, particularly in the crossover region, which plays a crucial role in determining the zero-shear viscosity (η_0_). Compared to other regression algorithms tested, the ANN model exhibits superior interpolation capabilities. However, accurate extrapolation to the Newtonian regime depends heavily on the quality and availability of the training input data, highlighting the need for further optimization in scenarios requiring precise extrapolation, particularly in high-viscosity systems.

Predictions were extended to conditions beyond those covered by the NEMD results, with CY fitting serving as a reference to rationalize prediction trends. Due to *n*-hexadecane’s crystallization tendency at certain conditions^44^ (e.g., 300 K and 300 MPa), the validity of our predictions may be restricted to conditions where thermodynamic forces dominate. Although indirect, the CY fitting method reliably captured the rate-dependent η, particularly the shear-thinning behavior at high γ̇ under specific *T* and *P*_*zz*_ conditions.^10^ To further validate the representation of CY fittings within the linear-response regime, particularly when the NEMD data are sparse near the crossover, zero-shear η_0_ derived from EMD simulations was used as a benchmark. As detailed in the SI, η_0_ from EMD simulations closely matched those obtained from CY fitting, affirming the latter’s validity as a reference. Leveraging these benchmarks, additional interpolated ML predictions were generated for new (η, *T*, *P*_*zz*_) conditions (Figure 3a,b, solid triangles), which adhere well to expected trends based on uniform intervals of condition variations. Beyond visual inspection, ML-predicted data effectively collapse onto a master curve (Figure 3c) when normalized as η̃ = η/η_0_ and $\overset{\sim }{\overset{˙}{\gamma }}=\mathrm{\gamma ̇}/{\mathrm{\gamma ̇}}_{0}$ , where η_0_ and γ̇_0_ were obtained from respective CY fittings, further demonstrating the consistency and reliability of the predictions.

**Figure 3:**
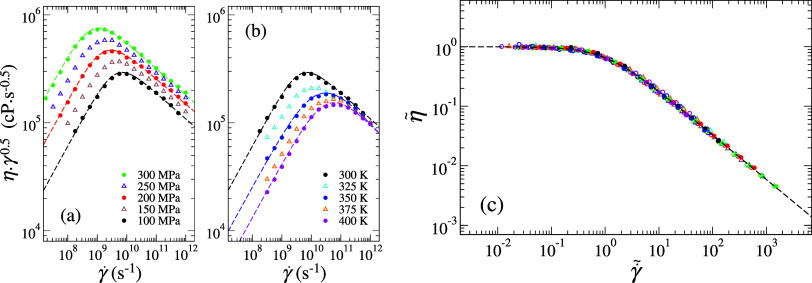
Intermediate scaling plot of machine learing (ML) predictions under interpolated conditions for (a) normal pressure (*P*_*zz*_) and (b) temperature (*T*), represented by hollow triangles. Dashed lines in (a, b) correspond to Carreau–Yasuda (CY) fits to the NEMD data for reference. (c) Collapse of all ML-predicted results (hollow symbols) and NEMD data (solid symbols) onto a master curve, expressed in terms of normalized viscosity (η̃) and normalized shear rate ( $\overset{\sim }{\overset{˙}{\gamma }}$ ). The dashed line represents CY fitting with η̃_0_ = 1 and ${\overset{\sim }{\overset{˙}{\gamma }}}_{0}=1$ . Error bars smaller than the symbol sizes are not shown.

Our supervised ANN model demonstrated exceptional performance in directly mapping physical parameters (γ̇, *T*, and *P*_*zz*_) to viscosity the target outputs, providing significant advantages in scalability and efficiency. Unlike unsupervised local dynamics ensemble (LDE)-based methods, this direct prediction (DP) approach requires less memory, eliminates molecular-type-specific adjustments, and simplifies implementation. While computationally efficient, the DP approach relies heavily on the quality and diversity of the training data set, posing potential challenges when extrapolating to conditions or molecular systems beyond the training range. The model’s predictions are grounded in well-defined physical parameters (γ̇, *T*, and *P*_*zz*_), making its behavior more transparent and easier to validate against known physical principles. Per our tests, incorporating additional input features, such as density (ρ) and radius of gyration (*R*_g_), did not significantly improve the prediction accuracy. However, these parameters could be valuable as output features, providing insights into molecular dynamics and temporal trends.

### Roles of *T* and *P*_*zz*_

The dependences of equilibrium viscosity η_0_ in the linear-response regime on *T* and *P*_*zz*_ have been explored in our previous work^10^ for the same linear alkane system. Notably, as *T* decreases, a transition from non-Arrhenius to Arrhenius behavior is observed, corresponding to a fragile-to-strong transition. Meanwhile, the dependence of η_0_ on *P*_*zz*_ follows a generalized hybrid function, where a power-law term dominates at low-to-negative *P*_*zz*_, while an exponential term governs at moderate-to-high *P*_*zz*_. However, in the shear-thinning regime at high γ̇, the dependences of η­(γ̇) on *T* and *P*_*zz*_ exhibit distinct trends: parallel for *T* (Figure 3a) and converging for *P*_*zz*_ (Figure 3b). The rate at which η­(*T*) and η­(*P*_*zz*_) decrease with increasing γ̇ is quantified by the shear-thinning exponent *n*, obtained from CY fitting. As illustrated in Figure 4, *n* remains nearly constant across different *P*_*zz*_ values but increases linearly with *T*. This highlights distinct dynamical behaviors and suggests that assuming a constant *n* or simply setting *n* to 0.5 under various conditions can lead to significant fitting errors.

**Figure 4:**
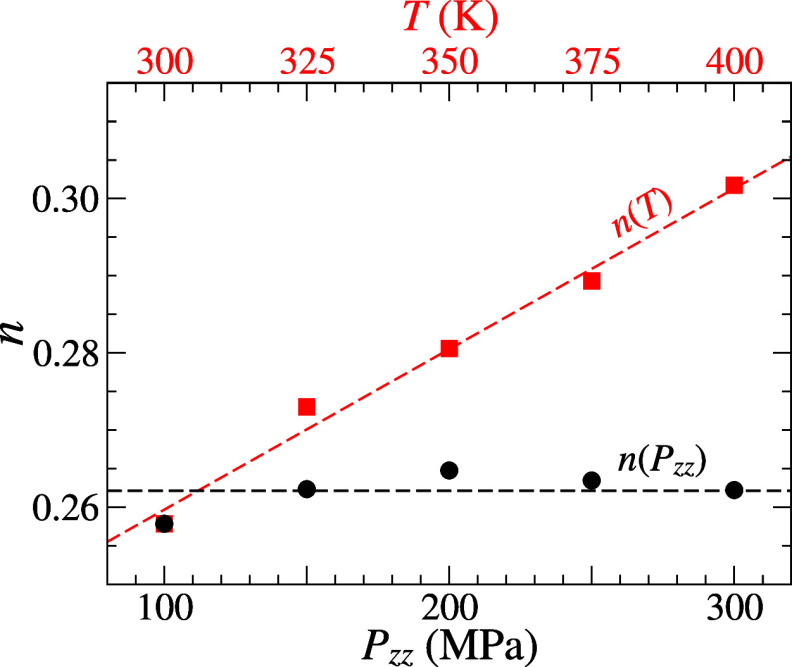
Variations of the shear-thinning exponent (*n*) obtained from Carreau–Yasuda (CY) fitting as functions of temperature (*T*, red) and normal pressure (*P*_*zz*_, black).

The trends observed in Figure 4 contrast with those predicted by the Carreau model,^45^ where *n* increases with *T* but decreases with *P*_*zz*_. Generally, increasing the temperature leads to larger vibrational amplitudes and thermally induced volumetric expansion, which weakens intermolecular interactions and facilitates molecular alignment under shear. In contrast, pressure primarily affects molecular proximity by increasing the density and modulating the corrugation barrier during shear. In the Newtonian regime, temperature effects typically dominate, as thermally induced volume change occurs more readily than those caused by external pressure due to the low compressibility of liquids. While *T* and *P*_*zz*_ influence η in a generally comparable manner,^10^ both often exhibit exponential or stretched-exponential behavior. At high γ̇ in the shear-thinning regime, externally applied forces induce intense atomic collisions, leading to a significant temperature rise. However, in molecular simulations, temperature is regulated via velocity rescaling, which artificially alters system dynamics while ensuring that the intrinsic response to shear remains dominant. Although removing thermal heating effects would clarify the individual contributions of *T* and *P*_*zz*_, this is practically impossible due to the limited thermal conductivity of solid counterfaces. This convergence persists in the shear-thinning regime as long as the system maintains consistent structural or phase responses, ensuring smooth variations in shear stress.^10^

In the shear-thinning regime, as γ̇ increases, liquid molecules tend to stretch in response to shear stresses, aligning their longitudinal direction parallel to the streaming direction. This morphological change facilitates sliding and can be characterized using the radius of gyration components:

$$R_{g}^{2}=\left\langle \frac{1}{M}\underset{i}{\sum }m_{i}{\left(r_{i}-r_{\mathrm{cm}}\right)}^{2}\right\rangle$$ 3

where *M* represents the total mass of a molecule, *r*_*i*_ and *r*_cm_ denote the positions of the *i*th monomer and the center of mass of the molecule, respectively. Unlike the layering-like structure observed in liquids confined to nanometer-scale slits,^26^ the spatially resolved density of bulk-phase liquid Couette flow remains constant even under relatively high normal pressure. As shown in Figure 5, structural anisotropy emerges when the system enters the shear-thinning regime, varying nonmonotonically with γ̇. The alignment of molecules parallel to the streaming direction (indicated by higher *R*_g,__*x*_^2^) reduces the number of atomic collisions, leading to a lower τ­(γ̇) that deviates from the trend. At high γ̇, the rapid expansion of molecular spacing, evidenced by the significant density drop shown in Figure 1b, causes molecules to coil up again. Further increasing γ̇ can induce a liquid-to-gas phase transition, where the three *R*_g_^2^ components converge and become comparable. While the dependences of *R*_g_(γ̇) on *P*_*zz*_ and 1/*T* appear similar at low γ̇, the temperature effect becomes less significant when *R*_g_^2^ exceeds the extreme values as γ̇ increases.

**Figure 5:**
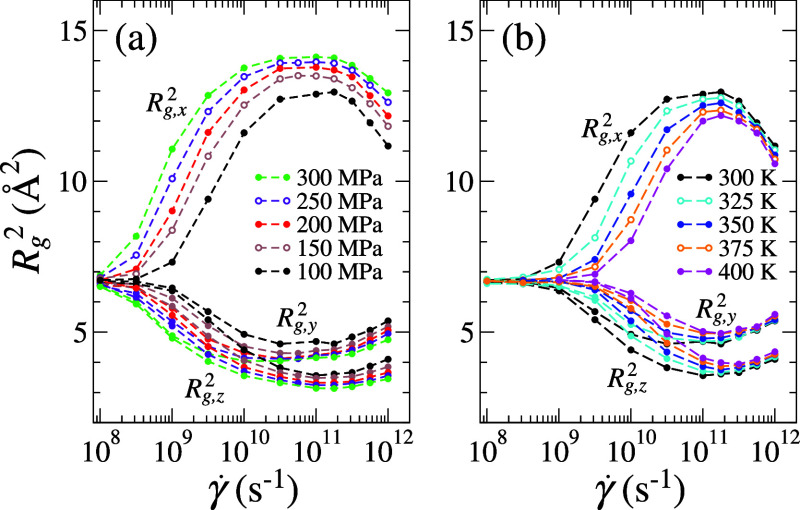
Radius of gyration components (*R*_g_^2^) as a function of shear rate (γ̇) under varying (a) normal pressure (*P*_*zz*_) at a constant temperature (*T*) of 300 K and (b) *T* at a constant *P*_*zz*_ of 100 MPa. Solid symbols represent *R*_g_ values obtained directly from NEMD simulations, while hollow symbols denote predictions from a separate machine learning (ML) model trained to predict *R*_g_ components. In this model, *R*_g_ values were also included as input features to leverage the internal correlations between structural descriptors. ML predictions are with 95% confidence intervals.

## Conclusions

This work establishes a comprehensive computational framework for predicting liquid shear viscosity (η) under coupled thermomechanical conditions (shear rate γ̇, temperature *T*, and normal pressure *P*_*zz*_) through the integration of nonequilibrium molecular dynamics (NEMD) and machine learning. The developed supervised artificial neural network (ANN) accurately predicts η­(γ̇, *T*, *P*_*zz*_) using exclusively NEMD-generated training data, effectively capturing the complex interrelationships between these variables without requiring supplementary molecular descriptors such as density or radius of gyration. While demonstrating robust performance within the studied parameter space, this implementation serves as a foundation for future enhancements: extension to more extreme thermodynamic conditions, generalization to diverse molecular systems (including branched alkanes and polar solvents), and improved accuracy across expanded parameter spaces. These developments will advance the framework into a versatile predictive tool for complex fluid behavior.

We highlight the importance of constant-pressure control in NEMD simulations, achieved through the implementation of a modified LAMMPS command, ‘fix npt/sllod’. This ensures an accurate representation of system dynamics under varying pressure conditions. Our results reveal distinct influences of *T* and *P*_*zz*_ on the shear-thinning regime: the shear-thinning exponent (*n*) from Carreau–Yasuda fitting remains constant across different *P*_*zz*_ but increases linearly with 1/*T* within the studied range. Additionally, the radius of gyration components exhibits nonmonotonic trends as a function of γ̇, reflecting molecular morphological changes driven by shear-induced alignment and volume expansion. Notably, temperature effects become less significant at higher γ̇, where shear-driven dynamics dominate the system’s response.

## Supplementary Material





## Notes

Modified LAMMPS source code for NPT-controlled shear flow in NEMD (ZIP)
Validation of the modified ‘fix npt/sllod’ implementation in LAMMPS, equilibrium viscosity (η_0_) calculations, detailed protocols for ANN training, model interpretation via permutation importance and SHAP analysis, uncertainty quantification, and comparative machine learning performance metrics (PDF)
