# Supplementary material for: Probing Rate-Dependent Liquid Shear Viscosity Using Combined Machine Learning and Nonequilibrium Molecular Dynamics
Source: J Chem Theory Comput. 2025 Jun 3;21(12):5838–44. doi: 10.1021/acs.jctc.5c00293 (PMC12199457; doi:10.1021/acs.jctc.5c00293)
Supplement: Supplementary file 2 [file ct5c00293_si_002.pdf]

Supporting Information: Probing Rate-Dependent Liquid Shear Viscosity using Combined Machine Learning and Non-Equilibrium Molecular Dynamics

Hongyu Gao,[*,†] Minghe Zhu,[†] Jia Ma,[‡,†] Marc Honecker,[†] and Kexian Li[‡]

†*Department of Materials Science & Engineering, Saarland University, Campus C6.3, 66123 Saarbrücken, Germany*

‡*School of Civil and Environmental Engineering, Changsha University of Science and Technology, Changsha 410114, PR China*

E-mail: hongyu.gao@uni-saarland.de

Phone: +49 681-302-57458

Validation of NPT Predictions

In our NEMD simulations, constant shear rates ($\dot{\gamma}_{\text{app}}$) were applied to mimic Couette flow, resulting in linear velocity profiles perpendicular to the shear plane. Figure S1 presents three representative cases with $\dot{\gamma}_{\text{app}} = 1.0\times10^{12}$, 3.2×10^{11}, and 1.0×10^{11} s^{-1}. The slope of each fitting curve ($\Delta v_x/\Delta z$) represents the actual shear rates ($\dot{\gamma}_{\text{act}}$), which closely align with the applied values with deviations of less than 1%.

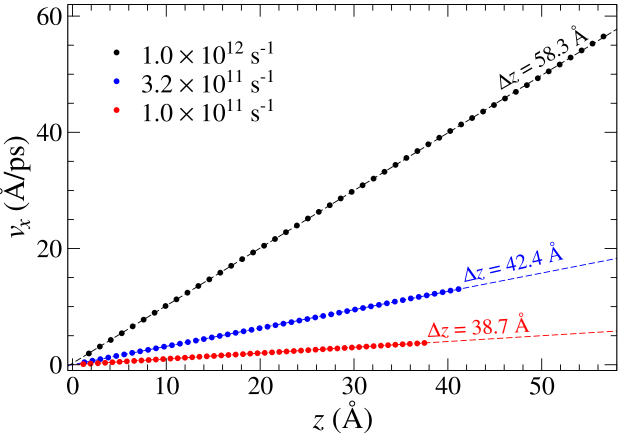

Figure S1: Representative velocity profiles perpendicular to the shear plane for three applied shear rates: $\dot{\gamma}_{\text{app}} = 1.0\times10^{12}$, 3.2×10^{11}, and 1.0×10^{11} s^{-1}, obtained from NEMD simulations. Here, v_x represents the spatially averaged velocities, and Δz denotes the average thickness of the liquid film. The simulations were performed under NPT/SLLOD conditions with T=400 K and P_{zz}=100 MPa.

As shown in Fig. S2, shear viscosity (η) predictions under const.-P_{zz} (NPT) control (red diamonds) closely matched those interpolated from const.-ρ (NVT) control. The results from the latter (hollow symbols) were fitted using Eq. 9 from our previous work.[S1] Variations in system dimensions under const.-ρ (NVT) control ensured that the target pressure range was adequately covered.

Predicting η_0 from EMD

To validate the extrapolation of NEMD results into the linear-response regime, the equilibrium viscosity (η_0) was derived from the fluctuation-dissipation theorem. In this approach,

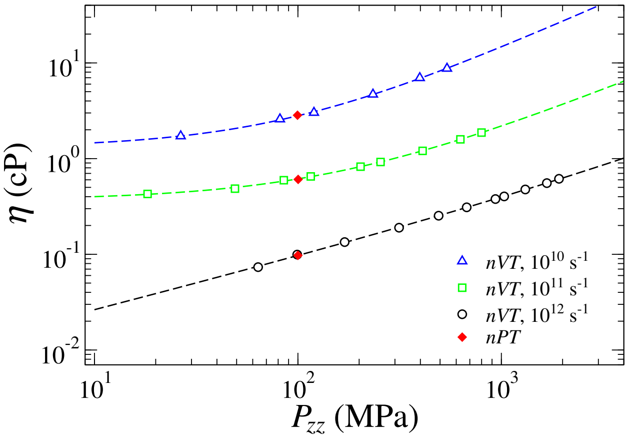

Figure S2: Comparison of NEMD-predicted shear viscosity (η) from const.-P_{zz} (solid symbols) and const.-ρ (hollow symbols) controls at shear rates ($\dot{\gamma}$) of 10^{10}, 10^{11}, and 10^{12} s^{-1}. Error bars are within the symbol sizes.

viscosity is calculated from the stress autocorrelation function (SACF), defined as:

$$C_{ij}(t) = \langle P_{ij}(t)P_{ij}(0)\rangle, \tag{1}$$

where $P_{ij}(t)$ represents the off-diagonal components of the stress tensor at time t, with $i \neq j \in \{x, y, z\}$. The SACF was computed using the fast Fourier transform (FFT), and the equilibrium viscosity was obtained by integrating the SACF over time according to the Green-Kubo formalism:[S2,S3]

$$\eta_0 = \frac{V}{k_\mathrm{B}T}\int_0^\infty C_{ij}(t)dt, \tag{2}$$

where V is the system volume, k_B is the Boltzmann constant, and T is the temperature. Equilibrium molecular dynamics (EMD) simulations were performed in the constant-volume (NVT) ensemble, with system dimensions pre-determined from constant-pressure (NPT) simulations to ensure that the mean hydrostatic pressure closely matched the target pressure (P_{zz}) applied in NEMD simulations (deviation$<$1%). The Nosé-Hoover[S4,S5] thermostat was employed to maintain the system temperature at the target value.

The actual correlation time (τ_c) used in the calculation of η_0 significantly exceeded the value estimated via the trapezoidal rule approximation (~2.5 ps), as shown in Fig S3a, to

ensure full capture of the stress response lag. To minimize noise, the total EMD simulation time was extended to at least 200 ns for each case. As illustrated in Fig. S3b, the extrapolated Newtonian viscosity at low shear rates (η_0) from NEMD closely aligns with the equilibrium viscosity (η_0^{EMD}) predicted by EMD. The NEMD data were fitted using the Carreau-Yasuda (CY)[S6,S7] model, as described in the main text. The close agreement between NEMD and EMD predictions, with differences of less than 5%, validates the reliability of NEMD data at low shear rates for use as training input and supports the adoption of CY-fitted curves as references for Newtonian viscosity.

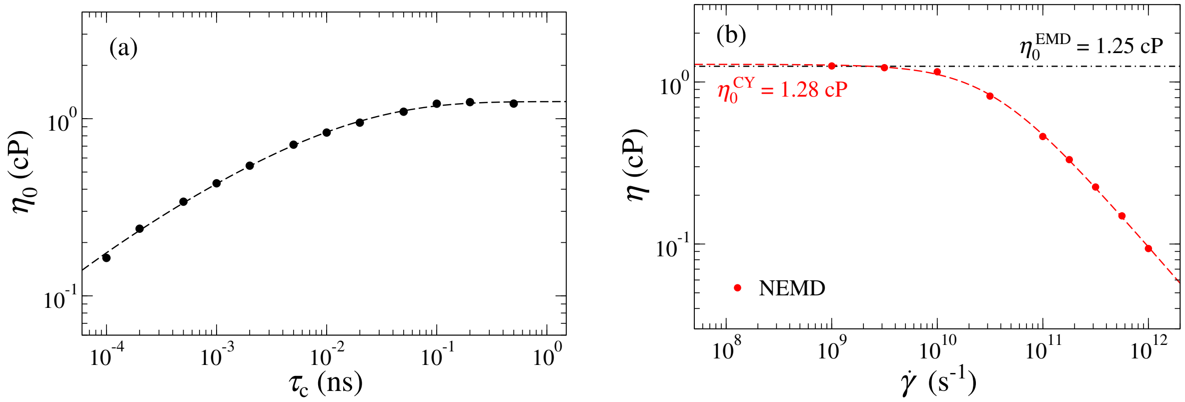

Figure S3: (a) Equilibrium viscosity (η_0) as a function of correlation time (τ_c) calculated using the EMD Green-Kubo method. The τ_c-independent η_0 (η_0^{EMD}) was determined once η_0 plateaued. (b) Comparison of NEMD-extrapolated Newtonian viscosity (η_0^{CY}) obtained via Carreau-Yasuda fitting with equilibrium viscosity (η_0^{EMD}) from EMD simulations. For both cases, P_{zz} in NEMD and hydrostatic pressure in EMD were set to 100 MPa, with temperatures maintained at 400 K.

ANN Training Protocol and Convergence Analysis

All input feature (shear rate, temperature, normal pressure) were normalized to [0, 1] range using scikit-learn's MinMaxScaler. This preprocessing step improved model convergence compared to unscaled features. Training employed early stopping through hyperparameter optimization (RandomizedSearchCV with 5-fold cross-validation), evaluating epoch counts from 100 to 5000. The optimal configuration (3000 epochs, batch size=16, Adam optimizer)

was selected when validation loss plateaued. Figure S4 shows the complete training history, demonstrating stable convergence with final losses of 3.4×10^{-4} (training) and 1.5×10^{-3} (validation).

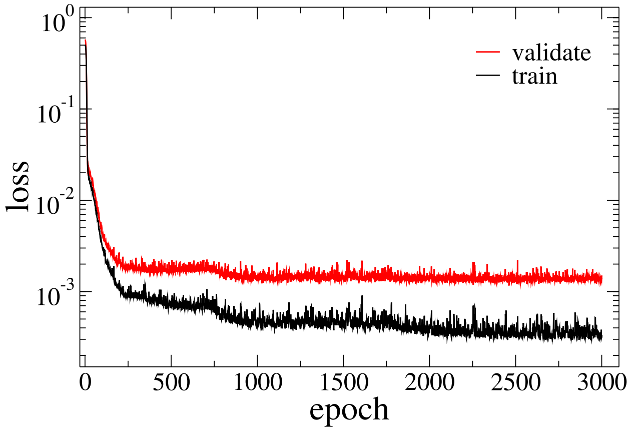

Figure S4: Training and validation losses from the best-performing ANN model (Adam optimizer, batch size of 16) across 3000 epochs.

Model Interpretation Analysis

Figure S5a presents permutation importance scores for normalized temperature (T), normal pressure (P_{zz}), and logarithmic shear rate ($\log(\dot{\gamma})$). The dominant influence of $\log(\dot{\gamma})$ (0.047 ± 0.003) is followed by moderate contributions from P_{zz} (0.012 ± 0.001) and T (0.008 ± 0.001). SHAP dependence plots (Fig. S5b-S5d) visualize the feature-specific impact on predictions: (b) pressure shows weak negative influence, (c) temperature induces an inverse Arrhenius-like effect, and (d) $\log(\dot{\gamma})$ reveals nonlinear shear-thinning trends. Negative SHAP values correspond to suppression of predicted viscosity.

Model Uncertainty Analysis

Figure S6 presents detailed uncertainty analysis using Monte Carlo dropout (50 forward passes per input). The panels show the distribution of 95% confidence interval widths as

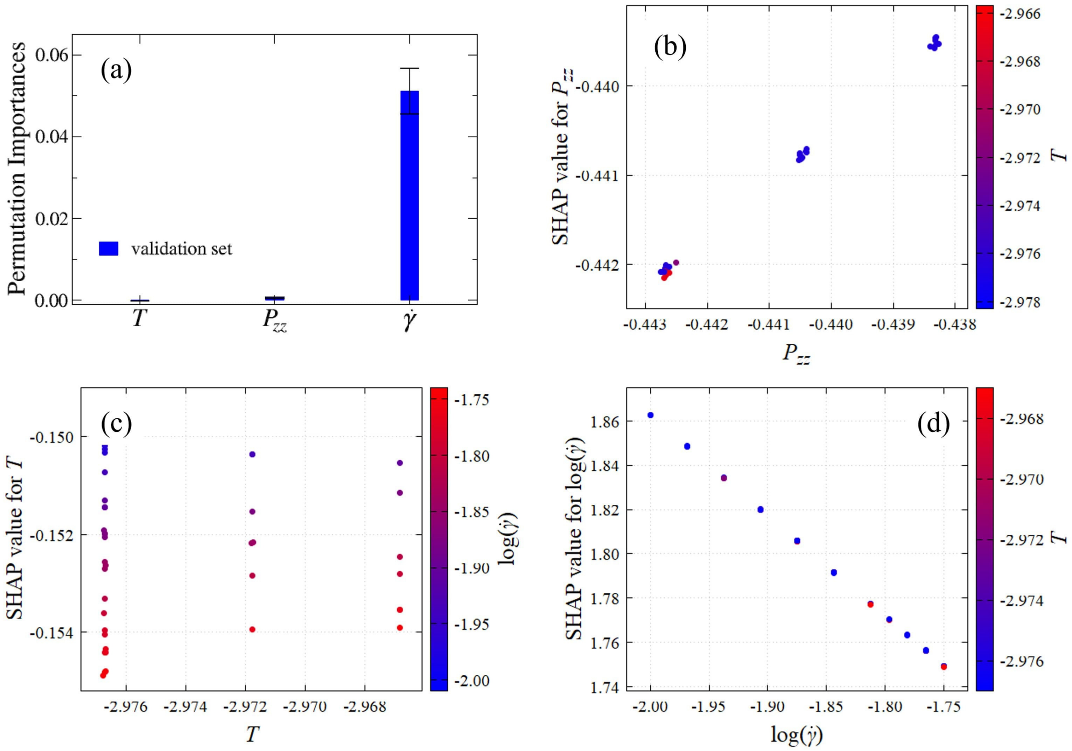

Figure S5: Model interpretation plots. (a) Permutation importance ranks feature contributions. (b–d) SHAP dependence plots reveal how individual features ($P_{zz}, T, \log(\dot{\gamma})$) influence predictions. Negative SHAP values indicate lower predicted outputs. Larger absolute SHAP values imply stronger feature influence.

functions of shear rate [$\log(\dot{\gamma})$], temperature (T), and normal pressure (P_{zz}). Uncertainty is highest near the shear-thinning crossover, at low T, and under elevated P_{zz}, reflecting both underlying physical complexity and data sparsity. These findings confirms that the model's confidence estimates are consistent with known rheological behavior.

Performance Comparison of ML Models for Viscosity Prediction

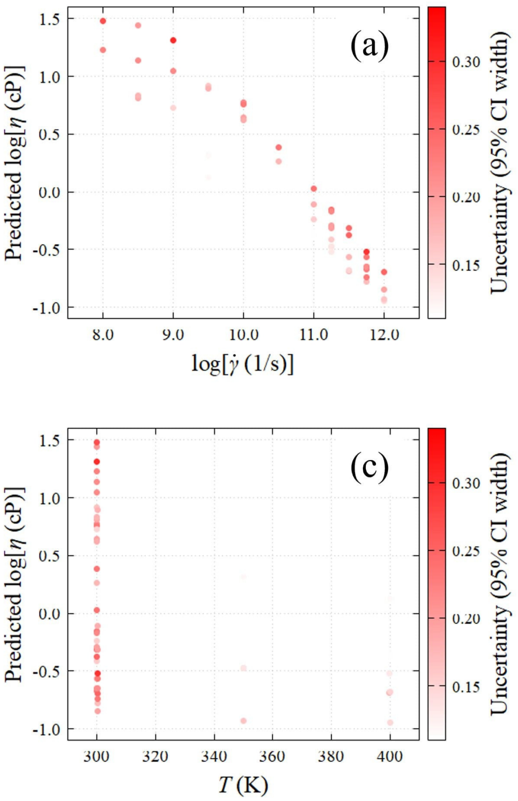

Figure S6: Feature-specific uncertainty analysis of viscosity predictions: Uncertainty vs. (a) shear rate [$\log(\dot{\gamma})$], (b) Temperature (T), and (c) normal pressure (P_{zz}). All panels display 95% confidence interval (CI) from Monte Carlo dropout (50 forward passes) with the same color scale.

Table S1: Performance metrics (MSE, MAE, R^2, RMSE) of representative machine learning models applied to viscosity prediction. Models evaluated include linear regression (LR), random forest (RF), extra trees (ET), gradient boosting (GB), hist gradient boosting (HGB), support vector regression (SVR), k-nearest neighbors (KNN), and artificial neural network (ANN). The ANN demonstrates superior accuracy across all metrics, supporting its selection as the final predictive model.

	LR	RF	ET	GB	HGB	SVR	KNN	ANN
MSE	5.19	1.52	3.35	7.18	1.55	1.21	1.55	0.00
MAE	1.36	0.64	0.78	1.31	0.72	0.56	0.56	0.01
R^2	0.48	0.84	0.66	0.28	0.84	0.87	0.84	0.99
RMSE	0.66	0.35	0.53	0.78	0.36	0.32	0.36	0.01

References

(S1) Gao, H.; Müser, M. H. On the Shear-Thinning of Alkanes. *Tribology Letters* **2023**, *72*, 16.

(S2) Green, M. S. Markoff Random Processes and the Statistical Mechanics of Time-Dependent Phenomena. II. Irreversible Processes in Fluids. *The Journal of Chemical Physics* **1954**, *22*, 398–413.

(S3) Kubo, R. Statistical-Mechanical Theory of Irreversible Processes. I. General Theory and Simple Applications to Magnetic and Conduction Problems. *Journal of the Physical Society of Japan* **1957**, *12*, 570–586.

(S4) Nosé, S. A molecular dynamics method for simulations in the canonical ensemble. *Molecular Physics* **1984**, *52*, 255–268.

(S5) Hoover, W. G. Canonical dynamics: Equilibrium phase-space distributions. *Phys. Rev. A* **1985**, *31*, 1695–1697.

(S6) Carreau, P. J. Rheological Equations from Molecular Network Theories. *Transactions of The Society of Rheology* **1972**, *16*, 99–127.

(S7) Yasuda, K.; Armstrong, R. C.; Cohen, R. E. Shear flow properties of concentrated solutions of linear and star branched polystyrenes. *Rheologica Acta* **1981**, *20*, 163–178.
